# How Divergent Views of Specialist Nurse Status Affect IQNs’ Skill Utilisation in Australia

**DOI:** 10.1155/jonm/5922358

**Published:** 2026-02-03

**Authors:** Chanchal Kurup, Adam Scott Burston, Vasiliki Betihavas, Elisabeth Ruth Jacob

**Affiliations:** ^1^ School of Nursing, Midwifery and Paramedicine, Faculty of Health Sciences, Australian Catholic University, Melbourne, Australia, acu.edu.au; ^2^ Central Queensland University, Bruce Highway, North Rockhampton, Queensland, Australia, health.qld.gov.au; ^3^ Nursing Research and Practice Development Centre, The Prince Charles Hospital, Chermside, Queensland, Australia, health.qld.gov.au; ^4^ School of Nursing and Midwifery, University of Notre Dame, Notre Dame, Australia, nd.edu

## Abstract

**Aim:**

To examine how internationally qualified nurses (IQNs) and Australian nurse managers conceptualise specialist nurse status within speciality nursing and how definitional divergence may contribute to the underutilisation of IQNs’ specialist skills.

**Design:**

A sequential, explanatory mixed‐methods study grounded in pragmatism.

**Methods:**

Phase 1 used a descriptive cross‐sectional survey (July–September 2022) distributed via social media, professional organisations and snowball sampling. Phase 2 involved qualitative data collection (December 2022–March 2023) through three focus groups with seven IQNs and eight individual interviews with nurse managers. Participants were asked to define specialist nurse status (survey prompt: ‘specialty nurse or clinical nurse specialist’), and data were analysed using content analysis (Phase 1 open text) and reflexive thematic analysis (Phase 2).

**Results:**

A total of 115 participants (71 IQNs; 44 nurse managers) provided definitional statements. Both groups drew on experience, education and advanced skills, but with different emphases. IQNs were significantly more likely than nurse managers to prioritise clinical experience as central to specialist nurse status (*χ* ^2^ (1, *N* = 115) = 7.94, *p* = 0.005, Cramér’s V = 0.26). No statistically significant difference was observed in references to education (*p* = 0.44). References to advanced skills and expanded scope were infrequent and differed in emphasis, with nurse managers more commonly highlighting leadership, mentoring and quality improvement functions, while IQNs more often emphasised clinical autonomy. These divergences indicate a recognition mismatch that may delay or limit deployment of IQNs into specialist roles.

## 1. Introduction

Registered nurses (RNs) constitute a significant portion of the global healthcare workforce, yet a persistent shortage of nurses continues to jeopardise healthcare systems worldwide [1]. This deficit has created critical gaps in care provision, ultimately compromising patient outcomes [2]. Recent estimates indicate that approximately 77% of developed countries face workforce shortages [3]. An additional nine million nurses and midwives will be required to meet the United Nations’ Sustainable Development Goals (SDGs) by 2030 [4].

The imbalance between supply and demand is driven by multiple factors, including ageing populations, rising healthcare needs postpandemic and workforce attrition [5]. Projections suggest that nearly one million RNs will retire by 2030, exacerbating the crisis [6]. Further compounding the issue, many nurses opt for reduced workloads or entrepreneurship over full‐time hospital shifts, diminishing the pool of available RNs [7]. To address these workforce gaps, many countries actively recruit internationally qualified nurses (IQNs) through various visa pathways [8]. In several major English‐speaking nations, IQNs represent a substantial proportion of the nursing workforce [9]. For example, in 2019, IQNs accounted for 35.3% of RNs in Australia, 33% in Singapore, 31.6% in Switzerland, 26.1% in Ireland, 24.4% in Canada and 21.9% in the United Kingdom (Organisation for Economic Co‐operation and Development) [10–14].

Beyond general nursing shortages, demand is increasing for nurses with specialist expertise in high‐acuity and complex‐care areas such as intensive care, cardiology, emergency and renal care [15–17]. While international recruitment can mitigate workforce gaps, IQNs frequently experience barriers to recognition and deployment of specialist skills in host systems, leading to underutilisation and career regression [18, 19]. Understanding how ‘specialist nurse’ status is conceptualised by IQNs and those who recruit them is therefore important to improving workforce integration and optimising health system capability.

## 2. Background

Speciality nursing has been established internationally for decades [20, 21],​ and specialist roles exist across diverse jurisdictions [22]; International Council of Nurses [23, 24]. For this study, speciality nursing refers to the clinical field (e.g., oncology, ICU), whereas a specialist nurse refers to an RN recognised as practising at an advanced level within that field. Internationally, pathways to specialist nurse recognition vary. In some health systems, specialist recognition is closely tied to postgraduate education and formal credentialing; in others, specialist status is primarily established through extensive experience, high‐acuity exposure and task‐shifted responsibility [25, 26].

IQNs often report difficulty in translating overseas specialist expertise into host‐country recognition frameworks, particularly where there is no standardised approach to assessing equivalence of experience‐based pathways [18, 27]. In Australia, role titles and criteria are not nationally standardised and vary by jurisdiction and industrial instruments. For example, some systems formally recognise a CNS title with explicit postgraduate and experience requirements, whereas others use alternative classifications and different combinations of education, competence and service requirements [28–30]. This variability can create ambiguity during recruitment and credential assessment, especially when IQNs’ prior specialist practice was achieved via experience‐based progression rather than credential‐driven models.

In light of these challenges, this study aims to examine how IQNs and Australian nurse managers conceptualise specialist nurse status within speciality nursing and how definitional divergence may contribute to the underutilisation of IQNs’ specialist skills.

## 3. The Study

### 3.1. Aim

The aim is to examine how IQNs and Australian nurse managers conceptualise specialist nurse status within speciality nursing and how definitional divergence may contribute to the underutilisation of IQNs’ specialist skills.

### 3.2. Objective

The objective is to explore whether differences in definitions of specialist nurse status are associated with underutilisation of IQNs’ specialist skills in Australia.

### 3.3. Research Question

What differences exist between IQNs and nurse managers in Australia regarding understanding the term ‘specialist nurse’?

## 4. Methods

### 4.1. Research Design

This study utilised a sequential explanatory mixed‐methods design, in which Phase 1 quantitative findings directly shaped the development of the qualitative instruments in Phase 2. Survey results, particularly the distribution of themes related to education, experience and advanced practice, were translated into specific probes and prompts for the focus groups and interviews. This structured sequencing allowed the qualitative data to explain and elaborate on the quantitative patterns, consistent with established explanatory mixed‐methods methodology.

### 4.2. Study Setting and Sampling

Participants were recruited using purposive and convenience sampling methods across two phases. In Phase 1, participants were recruited via targeted outreach on social media platforms and professional networks, including the Australian College of Nursing (ACN). Recruitment advertisements were posted in 112 relevant Facebook groups, such as Migrants in Australia, Skilled Migrants in Australia, Nurses in Australia and New Humans of Australia. Additionally, informal posts in professional discussion forums facilitated snowball sampling. These advertisements provided detailed study information, eligibility criteria and a survey link. To ensure eligibility, respondents confirmed their qualifications after reading a standardised introduction on the survey landing page. The Participant Information Letter was also accessible. Recruitment messages were renewed biweekly to maintain engagement. Nurse managers were recruited through direct professional email invitations to 178 hospitals and aged care facilities. The study was further promoted in 21 relevant social media groups and three professional organisations. In Phase 2, a convenience sampling approach was used. Participants from Phase 1 (IQNs and nurse managers) were invited to provide their contact details for follow‐up interviews. Participation in both phases was entirely voluntary.

#### 4.2.1. Inclusion and Exclusion Criteria

Two participant groups were included. IQNs were registered with the Australian Health Practitioner Regulation Agency (Ahpra), employed in Australian healthcare for at least 1 year, and possessing speciality skills acquired overseas. Nurse managers were Ahpra‐RNs in managerial roles who recruited IQNs. Initial screening questions ensured only eligible participants proceeded. Individuals not meeting these criteria were excluded.

#### 4.2.2. Instrument Development and Validation

This survey formed part of a larger study examining barriers and facilitators influencing IQNs’ utilisation of speciality skills in Australia (blinded for peer review). A self‐developed survey instrument was designed following a comprehensive literature review. It included six open‐ and closed‐ended questions for IQNs and nine for nurse managers. This paper focuses on a pivotal open‐ended question from that survey: ‘In your understanding, what is the definition of a speciality nurse or clinical nurse specialist?’

To strengthen content relevance and clarity, the draft survey underwent structured review by an expert panel with expertise in nurse migration, speciality nursing and nursing workforce research. Experts provided written feedback focused on item relevance, wording clarity and alignment with the study purpose. Revisions were made iteratively to improve comprehensibility and remove ambiguity. A quantitative content validity index (CVI) was not calculated because experts did not rate items using a numerical relevance scale; instead, consensus was established qualitatively through structured written feedback and iterative refinement. This approach strengthened face and content appropriateness while acknowledging that future survey development could incorporate formal CVI procedures.

#### 4.2.3. Sample Calculation

Phase 1 was designed to identify descriptive patterns in how IQNs and nurse managers conceptualise specialist nurse status and to inform purposive qualitative exploration in Phase 2. Because the quantitative phase was exploratory and not intended for hypothesis‐driven estimation, an a priori power calculation was not conducted. Instead, a pragmatic target of at least 100 participants was set to support stable descriptive estimates and enable preliminary group comparisons on key definitional elements. The achieved sample (*N* = 115) was adequate for descriptive purposes and to guide Phase 2 instrument development. Post hoc considerations indicate that nonsignificant comparisons (e.g., nonsignificant comparisons) may reflect limited power to detect smaller effects and should be interpreted alongside the qualitative explanatory findings.

#### 4.2.4. Data Collection

Data collection spanned two phases and employed both quantitative and qualitative methods. Phase 1, conducted between July and September 2022 via REDCap, remained open for 2 months and was completely anonymous. It included quantitative items (e.g., Likert‐type questions) and qualitative open‐ended questions to capture in‐depth responses. Phase 2, conducted between December 2022 and March 2023, involved in focus group discussions with IQNs and semistructured interviews with nurse managers. Insights from Phase 1, particularly the wide variation in how participants defined ‘speciality nurse’, directly informed the development of the Phase 2 discussion guide. The Phase 2 protocol centred on a single core question derived from the Phase 1 open‐ended survey item, with the group and interview environment enabling participants to elaborate, justify and debate their viewpoints in greater depth. The guiding question for both focus groups and interviews is shown in Table 1.

**Table 1:** Focus group and interview question.

Focus group questions
“In your understanding, what is the definition of a speciality nurse or clinical nurse specialist?” Follow‐up prompt: “Could you expand on that?”

Three focus group sessions with IQNs lasted an average of 90 min (1–2 h), while eight semistructured interviews with nurse managers via Microsoft Teams averaged 45 min (30–60 min). All sessions were audio‐recorded, professionally transcribed and reviewed for accuracy.

#### 4.2.5. Data Analysis

Data analysis was conducted in two sequential phases consistent with the explanatory mixed‐methods design. Phase 1 quantitative survey data were exported from REDCap, cleaned in Microsoft Excel and analysed using SPSS v26. Descriptive statistics (frequencies, means and standard deviations) were first generated to summarise participant characteristics and identify distributional patterns in how ‘speciality nurse’ was defined [31]. To examine whether IQNs and nurse managers differed significantly in the emphasis they placed on key definitional components (experience, formal education and advanced skills), chi‐square tests of independence were performed. Effect sizes were calculated using Cramér’s V to determine the strength of associations. Given the exploratory nature of the quantitative phase and the descriptive purpose of Phase 1, an a priori power calculation was not feasible. Instead, a post hoc estimation indicated that the comparison underlying the nonsignificant result was underpowered, with approximately 40%–55% power to detect a medium effect size at *α* = 0.05. This level of power is below the commonly recommended 80% threshold, and therefore, nonsignificant findings should be interpreted cautiously and in conjunction with the qualitative explanatory phase. This inferential analysis enabled us to assess whether observed differences reflected statistically meaningful divergence rather than descriptive variation alone.

Qualitative data from the open‐ended survey items and Phase 2 focus groups and interviews were analysed using a two‐stage approach. Open‐ended survey responses (Phase 1) underwent qualitative content analysis following the procedures of Jowsey et al. [32]. Meaning units were extracted, colour‐coded and grouped into preliminary categories. Two researchers independently coded the responses, and a third reviewer resolved discrepancies, ensuring rigour and consistency. Because many definitions contained more than one definitional element (e.g., experience and education), multiple codes could be applied to a single response; consequently, category totals may exceed 100%, and frequencies represent the proportion of participants who mentioned each element. These initial categories informed the development of the Phase 2 qualitative interview and focus group guides.

Phase 2 transcripts were analysed using Braun and Clarke [33] six‐step reflexive thematic analysis. This process involved familiarisation, generating initial codes, constructing and reviewing themes and synthesising final thematic interpretations. NVivo software supported systematic coding and theme development. Integration of the two phases occurred at the interpretation stage: qualitative themes were used to explain, contextualise and deepen the statistical patterns identified in Phase 1, providing the interpretive insight essential to the sequential explanatory design. Integration occurred through a joint display, where qualitative explanations were mapped to quantitative patterns to generate meta‐inferences. This analytic integration allowed authors to move beyond description and explore the cultural, structural and epistemological reasons underpinning group differences [34]. To preserve anonymity and enable traceability, participants were assigned identifiers at the point of transcription and data export. ‘IQN ##’ refers to a deidentified IQN respondent, and ‘Manager ##’ refers to a deidentified nurse manager respondent; numbers reflect the order of inclusion in the dataset and do not encode employer, location or seniority. The Phase 1 code ‘Advanced practitioner’ was treated as a subcode within the broader analytic category ‘Advanced skills/expanded scope’ to align Phase 1 coding with Phase 2 theme structure and the joint display logic.

#### 4.2.6. Ethical Considerations

Ethics approval was obtained from the university’s Human Research Ethics Committee (HREC) (approval number: [blinded for peer review]). In Phase 2, participants provided informed consent through a two‐step process. At the end of Phase 1, participants interested in follow‐up provided their contact details. A formal consent form was emailed before scheduling interviews or focus groups.

## 5. Results

A total of 115 participants, consisting of 71 IQNs and 44 nurse managers, were included in this study.

### 5.1. Participant Demographics

The gender distribution revealed that among the IQNs, 60 were female (84.5%), and 10 were male (14%), while among the recruiting managers, 30 were female (68.1%), and 12 were male (27.2%). One IQN (1.4%) and two recruiting managers (4.5%) preferred not to disclose their gender (Table 2).

**Table 2:** Demographics of IQNs and nurse managers.

IQNs *N = *71		Recruiting managers *N = *44	
Males	*n* = 10 (14%)	Males	*n* = 12 (27.2%)
Females	*n* = 60 (84.5%)	Females	*n* = 30 (68.1%)
Prefer not to say	*n* = 1 (1.4%)	Prefer not to say	*n* = 2 (4.5%)

**Age**		**Age**	

Mean	40.8	Mean	49.5
Range	18–64	Range	25‐over 65

The IQNs came from various countries, including India, the United Kingdom, the Philippines, Nepal, Nigeria, Pakistan, Vietnam, Singapore, South Africa, Ireland, Canada, Sri Lanka, the USA, Denmark, Germany and New Zealand. Recruiting managers were drawn from all states and territories of Australia.

Table 3 illustrates the distribution of participants by country of origin.

**Table 3:** IQNs and nurse managers, country of origin.

Country	IQNs (*n*)	IQNs (%)	Nurse managers (*n*)	Nurse managers (%)
Australia	0	0.0	24	54.5
India	34	47.9	3	6.8
United Kingdom	6	8.5	4	9.1
The Philippines	4	5.6	0	0.0
Nepal	4	5.6	0	0.0
Pakistan	2	2.8	0	0.0
Vietnam	2	2.8	0	0.0
Canada	2	2.8	0	0.0
Singapore	1	1.4	0	0.0
South Africa	1	1.4	0	0.0
Ireland	1	1.4	0	0.0
USA	1	1.4	0	0.0
Sri Lanka	1	1.4	0	0.0
Denmark	1	1.4	0	0.0
Germany	1	1.4	0	0.0
New Zealand	1	1.4	3	6.8
Nigeria	1	1.4	0	0.0
Not stated	8	11.3	10	22.7

### 5.2. Understanding of the Term ‘Specialist Nurse’

Participants’ definitional elements are summarised below in Table 4.

**Table 4:** Definitions of ‘specialist nurse’ by IQNs and nurse managers.

Category	IQNs (*n* = 71)^1^	IQNs (%)	Nurse managers (*n* = 44)	Nurse managers (%)	Analytic theme
Experience & expertise	39	54.9	13	29.5	Experience
Training/qualifications	21	29.6	14	31.8	Education & training
Knowledge/skills	10	14.1	5	11.4	Skills & scope
Specialised area focus	5	7.0	8	18.2	Skills & scope
Advanced practitioner (clinical autonomy)	4	5.6	0	0.0	Advanced skills/expanded scope^2^
Supports other RNs (teaching/mentoring/QI)	0	0.0	4	9.1	Advanced skills/expanded scope

^1^Because a single response could contain multiple elements, percentages represent the proportion of participants mentioning each element and totals can exceed 100%.

^2^Advanced skills/expanded scope represents a collapsed analytic category encompassing both clinical autonomy and leadership or system‐level functions described by participants.

#### 5.2.1. Experience and Expertise

Participants generally agreed that hands‐on involvement in specialised clinical settings was foundational to the concept of a speciality nurse. IQNs often stress the importance of direct, domain‐specific expertise, some describing it simply as ‘a registered nurse who is qualified and experienced in a particular speciality area’ (IQN 2). Others portrayed speciality nurses as those who have ‘experience in speciality wards like oncology and cardiology’ (IQN 39), underscoring the depth and breadth of clinical knowledge they gain from working in niche environments. Nurse managers echoed these sentiments, emphasising the operational demands of speciality units: ‘A nurse with experience in departments which handle speciality cases’ (Manager 7) and ‘a nurse with clinical experience in the nursing speciality’ (Manager 23). This collective emphasis on practical immersion underlines the perceived value of firsthand, sustained contact with specialised patient cohorts. Notably, a larger percentage of IQNs (54.9%, *n* = 39) who identified themselves as specialist nurses reported experience in an area as most important to be a specialist nurse, compared with only 29.5% (*n* = 13) of nurse managers.

#### 5.2.2. Education and Training

Formal qualifications were identified as required to be a specialist nurse by a large number of participants. IQNs noted that a ‘postgraduate university qualification in a speciality area’ (IQN 40) can be decisive in delineating a nurse’s advanced capacity. For some IQNs, thorough training also entails imparting knowledge effectively, describing a speciality nurse as ‘well trained and able to teach in their speciality’ (IQN 49). Nurse managers viewed advanced education as essential, favouring nurses who have ‘completed specialist training, preferably post‐grad, in cardiac care nursing’ (Manager 3). Moreover, they stressed that ‘these skills should be supported by an appropriate postgraduate qualification in the respective area’ (Manager 12). In this way, formal credentials were seen as a stamp of theoretical understanding and clinical skills. Notably, 31.8% (*n* = 14) of nurse managers reported higher education as important to be a specialised nurse, compared to 29.6% (*n* = 21) of IQNs.

#### 5.2.3. Advanced Skills and Scope

A final dimension of speciality nursing emerged around expanded practice and broader influence. IQNs regarded speciality nurses as ‘advanced practice’ professionals who ‘assume increased responsibility’ (IQN 47, Manager Interview 1) and ‘perform advanced practice nursing functions’ (IQN 35). This acknowledgment hints at an expectation of greater autonomy, leadership and decision‐making. Nurse managers highlighted mentorship, research and evidence‐based practice capabilities for their part: ‘A registered nurse with advanced practical skills and competency in teaching, mentoring, and evidence‐based care’ (Manager 12). One manager characterised a speciality nurse as ‘someone who has clinical expertise within a speciality and supports other RNs in their training assists in research or quality improvement, and acts as a leader/role model’ (Manager 5, IQN focus group 7). This broader outlook underscores how speciality nurses not only master clinical tasks but also guide teams, shape practice standards and contribute to organisational goals. A small difference was observed in references to advanced skills and expanded scope, with nurse managers more frequently highlighting leadership, mentoring and quality improvement functions (9.1%, *n* = 4) compared with IQNs’ emphasis on advanced clinical autonomy (5.6%, *n* = 4).

### 5.3. Statistical Comparison of IQN and Manager Definitions

To determine whether the observed differences between IQNs and nurse managers were statistically meaningful, chi‐square tests of independence were conducted for the three major definitional categories identified in Table 5: experience, education and advanced skills or expanded scope.

**Table 5:** Joint display table.

Theme	Qualitative findings	Quantitative findings	Mixed outcome	Meta‐inference
Experience & Expertise	‘Experience in oncology and cardiology defines a specialist’. (IQN 39) Managers: ‘A nurse with experience in departments that handle speciality cases’. (Manager 7)	IQNs: 39/71 (54.9%) Managers: 13/44 (29.5%) *χ* ^2^ = 7.94, *p* = 0.005	This is the only statistically significant divergence. IQNs rely on practice‐based, experiential definitions; managers acknowledge experience but do not treat it as central.	IQNs and nurse managers apply different recognition frameworks when defining specialist nurse status: IQNs prioritise experience‐based professional socialisation and clinical autonomy, whereas managers anchor recognition within Australian credentials, governance, and industrial structures. Although education and advanced skills are referenced by both groups, their meanings diverge, resulting in a misalignment that contributes to the underutilisation of IQNs’ specialist skills within the Australian healthcare system.
Formal Education/Postgraduate Training	IQNs: ‘Postgraduate qualifications help, but experience defines a specialist’. (IQN 40) Managers: ‘These skills must be supported by appropriate postgraduate study’. (Manager 12)	IQNs: 21/71 (29.6%) Managers: 14/44 (31.8%) *χ* ^2^ = 0.59, *p* = 0.44	Quantitatively similar frequency but qualitatively different meaning.	
Advanced Skills/Expanded Scope	IQNs: ‘Advanced practitioners assume increased responsibility’. (IQN 47) Managers: ‘Supports other RNs, mentors and leads quality improvement’. (Manager 5)	Low‐frequency descriptive difference; qualitative divergence in meaning rather than statistically reliable difference.	Conceptual mismatch in what ‘advanced’ implies.	

#### 5.3.1. Experience

IQNs were significantly more likely than nurse managers to emphasise clinical experience as a defining element of specialist nurse status (54.9% vs. 29.5%). The chi‐square analysis confirmed this difference as statistically significant, *χ* ^2^ (1, *N* = 115) = 7.94, *p* = 0.005, with a Cramér’s V of 0.26, indicating a small to medium effect size. This finding demonstrates that experiential mastery represents a key point of divergence between the two groups, with IQNs placing substantially greater emphasis on practice‐based expertise than nurse managers.

#### 5.3.2. Education

No statistically significant difference was observed between IQNs and nurse managers in the likelihood of emphasising formal postgraduate education as a defining component of specialist nurse status (29.6% vs. 31.8%). The chi‐square test indicated no meaningful group difference, *χ* ^2^ (1, *N* = 115) = 0.59, *p* = 0.44, with a Cramér’s V of 0.07, suggesting a negligible effect size. Although education featured prominently in both groups’ definitions, this finding indicates that IQNs and nurse managers did not differ substantially in how frequently they referenced formal education when conceptualising specialist nursing.

#### 5.3.3. Advanced Skills/Expanded Scope

Nurse managers were slightly more likely than IQNs to reference advanced skills and expanded scope of practice as defining elements of specialist nurse status (9.1% vs. 5.6%); however, frequencies were low, and no statistically reliable difference was demonstrated. This pattern suggests a modest difference in emphasis that should be interpreted cautiously and considered alongside the qualitative explanatory findings.

#### 5.3.4. Mixing of the Data

Joint display table is as follows.

## 6. Discussion

This mixed‐methods study examined how IQNs and Australian nurse managers conceptualise specialist nurse status within speciality nursing and how definitional divergence may contribute to the underutilisation of IQNs’ specialist skills. The integrated findings demonstrate that differences between the two groups are not merely semantic but reflect distinct recognition frameworks that shape how specialist expertise is assessed, valued and deployed. IQNs primarily draw on experience‐based professional socialisation and clinical autonomy to define specialist practice, whereas nurse managers situate recognition within Australian credentials, governance and industrial structures. These competing frameworks provide a clear explanatory lens for understanding the persistent underutilisation of IQNs’ specialist skills within the Australian healthcare system.

### 6.1. Experience and Expertise

Clinical experience emerged as the most significant point of divergence between IQNs and nurse managers. IQNs were significantly more likely to define specialist nurse status through experiential mastery, reflecting professional socialisation in health systems where sustained exposure, procedural autonomy and responsibility accumulation confer specialist recognition. In contrast, while nurse managers acknowledged the importance of experience, it was afforded less definitional weight within Australian credential‐based recognition structures, reinforcing a hierarchy in which experience alone is insufficient for specialist classification.

This divergence mirrors international evidence showing that nurses trained in many low‐ and middle‐income country contexts commonly achieve specialist responsibilities through high‐acuity rotations and task‐shifting models that extend nursing scope well beyond traditional Western boundaries [35, 36]. Australian practice environments, however, rely more heavily on postgraduate study, standardised competency frameworks and industrial classification systems to validate specialist status [37]. As a result, accumulated international expertise may not translate directly into locally recognised specialist roles.

The structural consequences are substantial. IQNs with extensive specialist experience may be placed in generalist positions or required to repeat qualifications already mastered overseas, delaying progression and undermining professional identity [38]. This pattern contributes to reduced self‐efficacy and increased turnover intention, consistent with broader migration literature [39]. Although both groups value experience, education and skill, their ranking of these elements differs, leading to conflicting assumptions about what constitutes specialist practice.

### 6.2. Formal Education and Postgraduate Training

Education was referenced at similar frequencies by both groups; however, its function within each recognition framework differed markedly. For nurse managers, postgraduate education operates as a formal gatekeeping mechanism embedded within accreditation standards, enterprise bargaining agreements and regulatory requirements. For IQNs, education is viewed as additive to already established specialist expertise rather than the foundation of specialist identity.

This divergence explains why comparable quantitative emphasis on education does not translate into shared expectations regarding specialist readiness. In many source countries, structured postgraduate pathways are inconsistently available or not required for assuming advanced clinical responsibilities [35, 36, 40]. Consequently, IQNs may enter the Australian workforce with substantial experiential expertise but without the specific local credentials required for specialist recognition. Reliance on qualification‐centric models without robust mechanisms to assess experiential equivalence risks overlooking highly capable practitioners and perpetuating role mismatch and dissatisfaction [41, 42].

### 6.3. Advanced Skills and Expanded Scope

Differences in conceptualising advanced skills further reinforced the recognition mismatch identified in the integrated findings. IQNs predominantly framed advanced practice in terms of clinical autonomy and expanded responsibility, reflecting practice environments in which nurses independently manage complex care due to resource constraints or intentionally broadened scopes of practice [23, 43]. In contrast, nurse managers are more frequently associated with advanced practice with leadership, mentoring, governance and quality improvement functions, aligning with Australian role expectations and CNS/CN frameworks [20, 44].

Although references to advanced skills and expanded scope were infrequent, the qualitative data reveal a clear conceptual divide. IQNs who have practised autonomously in high‐acuity settings may find themselves restricted to narrower scopes of practice within Australian healthcare systems, while nurse managers may interpret the absence of locally recognised leadership or governance indicators as a deficit in advanced practice readiness. This misalignment can prolong pathways to specialist recognition and delay optimal workforce deployment, limiting the effective utilisation of existing specialist capability [45].

### 6.4. Structural Misalignment and Skill Underutilisation

Taken together, the findings indicate that IQN specialist skill underutilisation is driven primarily by structural misalignment rather than individual capability deficits. Experience‐based international pathways to specialist practice do not map cleanly onto Australia’s credential‐centred recognition systems. When experiential expertise, additive education and clinically focused advanced practice are not translated into locally recognised specialist criteria, IQNs are more likely to be channelled into generalist roles despite possessing specialist capability.

This underutilisation has tangible system‐level consequences. Delayed recognition of specialist skills contributes to inefficient workforce deployment, increased burden on existing specialist staff and missed opportunities to enhance patient care in high‐acuity settings [44, 45]. In a healthcare system facing sustained specialist shortages, failure to harness available expertise represents a loss of clinical capacity and economic value [46].

In Australia, these challenges are compounded by jurisdictional variability in industrial classifications and role requirements. Differences across states in credential expectations, titles and recognition processes create inconsistent and unpredictable pathways for IQNs, even when overseas roles involved advanced clinical responsibility and leadership [47, 48]. Similar difficulties are reported internationally, where credential‐based models frequently fail to accommodate experience‐defined specialist pathways, amplifying professional and emotional strain for migrating nurses [49, 50].

### 6.5. Implications for Policy and Practice

Addressing specialist skill underutilisation requires a shift from credential‐only recognition toward competency‐informed assessment. Strengthening Recognition of Prior Learning (RPL) mechanisms, developing speciality‐specific bridging pathways and enhancing managerial understanding of international training models would enable more accurate assessment of specialist capability. Evidence from simulation‐based education demonstrates that patient safety competencies, such as clinical judgement, communication, escalation of care and risk management, can be reliably developed and assessed through structured simulation designs, even in preregistration contexts [51]. Such approaches would support faster deployment of IQNs into appropriate roles, reduce attrition and improve patient care outcomes while strengthening workforce resilience [41, 52].

### 6.6. Strengths and Limitations of the Work

This study integrated quantitative and qualitative methods to provide a nuanced examination of specialist nurse recognition from both IQN and nurse manager perspectives. Reflexivity was actively maintained, with the lead researcher engaging in structured self‐reflection to minimise bias [53]. Expert review of survey items and triangulation across analysts strengthened interpretive rigour.

Limitations include reliance on online recruitment, which may have restricted participation and introduced response bias [54, 55]. Public distribution methods may have disproportionately attracted participants with negative experiences, while managers from organisations with effective IQN integration may have been underrepresented. Some nurse managers were themselves immigrant nurses, offering valuable dual perspectives but also potential interpretive complexity.

Future research should examine the longitudinal impact of specialist skill underutilisation on IQN career trajectories, retention and patient outcomes, and evaluate alternative accreditation and employer‐led recognition models.

## 7. Conclusion

The findings of this study demonstrate that IQNs’ specialist skills are frequently underutilised, not due to lack of expertise, but because experience‐based professional socialisation does not align neatly with Australian credential and governance frameworks. Divergent recognition logics between IQNs and nurse managers contribute to delayed or denied specialist recognition, resulting in workforce inefficiency and professional stagnation. Implementing competency‐informed recognition processes, speciality‐focused bridging pathways and improved understanding of international specialist training routes is essential to enable IQNs to practise at their level of expertise and to strengthen Australia’s specialist nursing workforce.

## Author Contributions

Conceptualisation, methodology, formal analysis, investigation, writing–original draft preparation and writing–review and editing: Chanchal Kurup.

Conceptualisation, methodology, writing–review and editing and supervision: Adam Scott Burston, Vasiliki Betihavas and Elisabeth Ruth Jacob.

The authors have checked to ensure our submission conforms as applicable to the Journal’s statistical guidelines.

## Funding

No funding was received for this manuscript. Open access publishing was facilitated by the Central Queensland University, as part of the Wiley–Central Queensland University agreement via the Council of Australian University Librarians.

## Ethics Statement

Permission from the ACU’s Human Research Ethics Committee (HREC) was obtained (blinded for peer review) prior to the research.

## Consent

No patients were involved in this research study; therefore, patient consent was not required.

## Conflicts of Interest

The authors declare no conflicts of interest.

## Data Availability

The data that support the findings of this study are available on request from the corresponding author. The data are not publicly available due to privacy or ethical restrictions.
